# 

**Published:** 1884-10

**Authors:** 


					American Academy of DentalScience 279
AMERICAN ACADEMY OF DENTAL SCIENCE.
The 17th Annual Meeting of the American Academy of
Dental Science will be held in Boston on Wednesdays
November 5th, at 3 o'clock P. M.
The Annual address will be delivered by E. M. Harris.
D. D. S., of Boston,
E. E. HOPKINS,
Recording Secretary,
85 Newbury St., Boston.
MARYLAND STATE DENTAL ASSOCIATION.
The Second Annual Meeting of the Maryland State
Dental Association, will be held in Chemical Hall, Univer-
sity of Maryland, November 13th, 1884, 8 o'clock P. M.
WM. A. MILLS, Recording Secretary,
49 S. Broadway, City.
To Readers and Correspondents
Communications intended for publication, and exchange journals and
papers should be addressed to the Editor of the American Journal of
Dental Science, 259 N. Eutaw St., Baltimore, Md. All letters relating
to the business of the Journal, or containing cash remittances, to be
directed to Messrs. Snowden & Cowman. Articles for publication should
be received by the editor at least three weeks previous to the first month
on which the number in which it is designed for them to appear, is issued-
|5F~ The American Journal of Dental Science will contain fifty
pages of reading matter in each number, making a volume of about
Six Hundred pages,
EXCLUSIVE OF ADVERTISEMENTS.

				

